# Prevalence and risk factors for bone loss in Southern Chinese with rheumatic diseases

**DOI:** 10.1186/s12891-020-03403-1

**Published:** 2020-06-30

**Authors:** Zhuoran Hu, Shuiming Xu, He Lin, Weifeng Ni, Qingyuan Yang, Jun Qi, Keqian Du, Jieruo Gu, Zhiming Lin

**Affiliations:** 1 Division of Rheumatology, Third Affiliated Hospital of Sun Yat-sen University, 600 Tianhe Road, Tianhe District, Guangzhou, 510630 China; 2 Division of Rheumatology, Ganzhou Municipal Hospital, Ganzhou, 341000 China; 3 Division of Rheumatology, Fujian Provincial Hospital, Fuzhou, 350000 China; 4 Division of Rheumatology, Second Affiliated Hospital of Shantou University Medical College, Shantou, 515000 China

**Keywords:** Rheumatic patients, Bone mineral density, Osteopenia, Osteoporosis

## Abstract

**Backgroud:**

This study is to explore the prevalence of different stages of bone loss and the potential risk factors in rheumatic patients.

**Method:**

A cross-sectional study recruits 1398 rheumatic patients and 302 healthy subjects. Demographic data, blood, and bone mineral density (BMD) tests are collected. Risk factors for bone loss in rheumatic patients are analyzed by logistic regression.

**Results:**

(1) Rheumatic patients are consisted of 40.0% rheumatoid arthritis (RA), 14.7% systemic lupus erythematosus (SLE), 14.2% osteoarthritis (OA), 9.2% ankylosing spondylosis (AS), 7.9% gout, 7.0% primary Sjogren syndrome (pSS), 3.8% systemic sclerosis (SSc), and 3.2% mixed connective tissue disease (MCTD). (2) In male patients aged under 50 and premenopausal female patients, the bone mineral density score of AS (53.9%, *P <* 0.001) and SLE (39.6%, *P* = 0.034) patients is lower than the healthy controls (18.2%). (3) Osteopenia and osteoporosis are more prevailing in male patients aged or older than 50 and postmenopausal female patients with RA (*P* < 0.001), OA (*P =* 0.02) and SLE (*P* = 0.011) than healthy counterparts. (4) Those with SLE, RA and AS gain the highest odd ratio of ‘score below the expected range for age’, osteopenia and osteoporosis, respectively. (5) Age, female, low BMI and hypovitaminosis D are found negatively associated with bone loss. Dyslipidemia and hyperuricemia could be protective factors.

**Conclusion:**

Young patients with AS and SLE have a significant higher occurrence of bone loss, and older patients with RA, OA and SLE had higher prevalence than healthy counterparts. SLE, RA, SSc and AS were founded significant higher risks to develop into bone loss after adjustment. Age, BMI and gender were commonly-associated with bone loss in all age-stratified rheumatic patients. These findings were not markedly different from those of previous studies.

## Background

Osteoporosis (OP) is a skeletal disease that refers to the reduction of bone mass and the deterioration of microstructure of bone tissue and leads to an increased risk of bone fragility and fracture and consequently, disability and mortality. Older age, low body mass index (BMI, kg/m^2^), female and post-menopause, smoking, vitamin D deficiency [1–3] have been proved to be generally and strongly related to OP and osteoporotic fracture. Rheumatic diseases (RD), including arthritis, diffuse connective tissue diseases, spondyloarthropathies, etc., are proved to be relevant to bone loss [4–13]. Disease-specific causes of secondary OP are well-established and shared in RD, like inflammation-associated osteoclast activation [14, 15], routine glucocorticoid (GC) treatment [16–19], and reduced physical activity, which in turn leads altered bone metabolism (favoring bone resorption) [20, 21] due to musculoskeletal pain and weakness. Also, disease activities would inhibit intestinal calcium and vitamin D absorption. In fact, chronic, systemic, or local inflammation and/or exposure to GC treatment cause an imbalance between bone formation and bone resorption [22] and which are both important determinants of bone loss in RD. Hence, rheumatic patients are more likely to suffer from osteoporosis. Nevertheless, more factors need to be included to explore the association. Levels of inflammatory markers, alcohol intaking, and medical history (e.g. diabetes, hypertension, dyslipidemia, and hyperuricemia) may play a role in the decreased bone mineral density (BMD).

Many studies have reported on the prevalence of different severities of bone loss in rheumatic patients [5, 10, 19, 23, 24]. However, the results are often presented in the form of one specific disease type comparing with RA and healthy subjects, such as rheumatoid arthritis (RA), systemic lupus erythematosus (SLE) and systemic sclerosis (SSc), instead of as a general rheumatic population. In 2016, a cross-sectional study in the South Korean reported the frequency of OP in the RA population was 46.8% [25]. In 2017, a Canada retrospective study revealed the occurrence of ‘score lower than expected range for age’, osteopenia and OP among 286 patients with SLE was 17.3, 12.3 and 43.2%, respectively [23]. A French comparative study enrolled 71 patients with SSc and 139 patients with RA showed a high prevalence of OP (30%), was increased compared with healthy controls and similar to RA group (32%) [6].

There is still insufficient data on the general prevalence of combining with bone loss in diverse rheumatic diseases with a large sample size in China. Almost studies published already were about a single disease. For increasing physician’s awareness of bone loss in rheumatic patients so as to improve early diagnosis in order to ease the social economic burden, we primarily sought to determine the prevalence of the impaired bone mass in patients with rheumatism and further investigate the potential risk factors by conducting a cross-sectional survey in four hospitals in different districts in Southern China: Third Affiliated Hospital of Sun Yat-sen University, Ganzhou Municipal Hospital, Fujian General Hospital, and the Shantou Second General Hospital. The principal center was the Third Affiliated Hospital, Sun Yat-sen University.

## Methods

### Study design, sample size and population

An analytical cross-sectional study design was carried out, and rheumatic in-patients were consecutively recruited considering individual classification criteria from the rheumatism departments in four hospitals from May 2017 to August 2018. We also contemporarily recruited healthy subjects who were free from rheumatic diseases and selected randomly from applicants for health checks in the same hospital. The ethical approval was obtained from the Ethics Committee of the Third Affiliated Hospital, Sun Yat-sen University, and all participants provided informed consent for publication of their clinical details. Patients who were diagnosed with (1) rheumatoid arthritis (RA); (2) osteoarthritis (OA); (3) systemic lupus erythematosus (SLE); (4) systemic sclerosis (SSc); (5) ankylosing spondylosis (AS); (6) primary Sjogren syndrome (pSS); (7) gout; (8) mixed connective tissue disease (MCTD); we also excluded (1) pregnant; (3) with malignant tumor and/or receiving chemotherapy; (4) aged under 18; (5) refusing to write informed consent. A systematic sampling design was used to select the participants. The sample sizes were estimated by PASS 15 software (https://www.ncss.com), with the statistical power (1-β) set 0.90, type I error (α) set 0.05 and assuming that the prevalence of complicating with OP was 35% among rheumatic patients and 20% [26] among healthy controls. The software calculated that a total sample size of at least 1653 would suffice. To ensure adequate events of each group, we finally recruited 1398 patients and 302 healthy controls (HC), totally 1700 participants for this study.

### Data collection, procedures, and tools

A standardized five-part questionnaire was designed to collect data. The first part of this questionnaire contained demographic information such as age, gender, height, weight, menopausal status, etc. The second part focused on medical history, diabetes mellitus (type 2), hypertension (primary or secondary), dyslipidemia and hyperuricemia. Part three consisted of the patient’s lifestyle habits including drinking and smoking and medication history. All variables in part two were dichotomous except conventional disease-modifying antirheumatic drugs (cDMARDs), which was an ordinal one; and part four of the questionnaire consisted of biochemical examinations. Detailed results of BMD test were recorded in the last part of the questionnaire.

The procedures of collection were in two steps. Participants filled in the first part of the questionnaire after admission. The other parts were completed by the trained physician according to the patients’ medical records or the HC reports after the patient had finished the blood test and BMD test at the same hospital.

### Blood samples and DXA tests

Blood samples were analyzed by standard laboratory techniques at the participating hospitals. Fresh blood samples were collected from each patient after the patient had been admitted, included detailed concentrations of blood calcium, serum phosphate, serum 25(OH)D3, serum creatine (sCr) and serum uric acid (sUA), c-reactive protein level (CRP), erythrocyte sedimentation rate (ESR) and plasm complement component 4 (C4). Blood lipid examination was also performed with no detail showing in our study but finally diagnosis.

Statistics After the blood samples had been taken, the patients were taken to the nuclear medicine department for bone mass density then assessed by dual-energy X-ray absorptiometry (DXA; Hologic Discovery A densitometer, Badford, MA, USA) at the lumbar spine L2 ~ L4(anterior-posterior view), femoral neck and total hip.

### Definitions

Body mass index (BMI) was calculated by dividing body weight by the square of height in meters (kg/m^2^). According to the definition of by WHO, BMI was categorized as underweight, normal, overweight and obese in the Chinese population when the individual had a BMI of < 18.5, ≥18.5 – < 24, and ≥ 24 – < 28, ≥28 respectively [27, 28]. Cigarette and alcohol consumption were further described as former/current smokers and non-smokers; regular or never/seldom drinking.

Medication history of participants defined as follow: (1) those who have consecutively taken orally or took GC ≥3 months [18] in the last 1 year before the day of BMD examination were ‘former or current chronic therapy of oral GC’; (2) those who had a history of consecutively taking cDMARDs ≥1 months, or used biological DMARDs (bDAMRDs) in the last 1 year were cDMARDs and/or bDMARDs users; (3) those has regularly taken NSAIDs ≥1 month was ‘NSAIDs user’.

BMD was expressed in standard deviation (SD) from the mean of healthy age- and sex-matched people (the *Z*-score) and as the number of SD from the mean of healthy, young sex-matched people (the *T*-score). All procedures were performed in accordance with the manufacturer’s standardized analysis software for hip and spine BMD measurements. *T*-score is recommended for males ≥50-year-old and postmenopausal women, but *Z*-score is preferable for males < 50-year-old and premenopausal women. Corresponding *T*-score or *Z*-score of each detective site was evaluated separately, but the lowest value of BMD in these measured sites was used. Result met the WHO classification [29] and the 2005 International Society for Clinical Densitometry (ISCD) [30] official positions.

### Data processing and statistical analysis

Data were entered into Microsoft Office Excel (version 2016), and then two of the physicians rechecked and transferred this data to the R software (version 3.6.1) for analysis. Descriptive statistics for continuous variables included means and standard deviation (with normal distribution) and medians and interquartile ranges (with non-normal distribution), while categorical variables are presented as frequency and percentage. Group comparisons between the rheumatic patients and the healthy subjects were performed by Student’s two-tailed *t*-test for normally distributed continuous variables and *Kruskal-Wallis H* test for non-normally distributed ones. Pearson’s chi-square test or Fisher’s exact test was performed for categorical variables and Cochran-Armitage trend test for ordinal variables as appropriate. To determine the association between impaired BMD and rheumatic diseases and potential risk factors, we conducted logistic regression analyses to calculate the odds ratios (OR) and corresponding 95% confidence intervals (95% CI). A *P-*value < 0.05 was considered statistically significant. No imputations of missing values were performed. Comparison analyses were carried out by using R-3.6.1 for windows, package ‘compareGroups’ version 4.1 [31].

## Results

### Baseline characteristics of patients

**Fig. 1:**
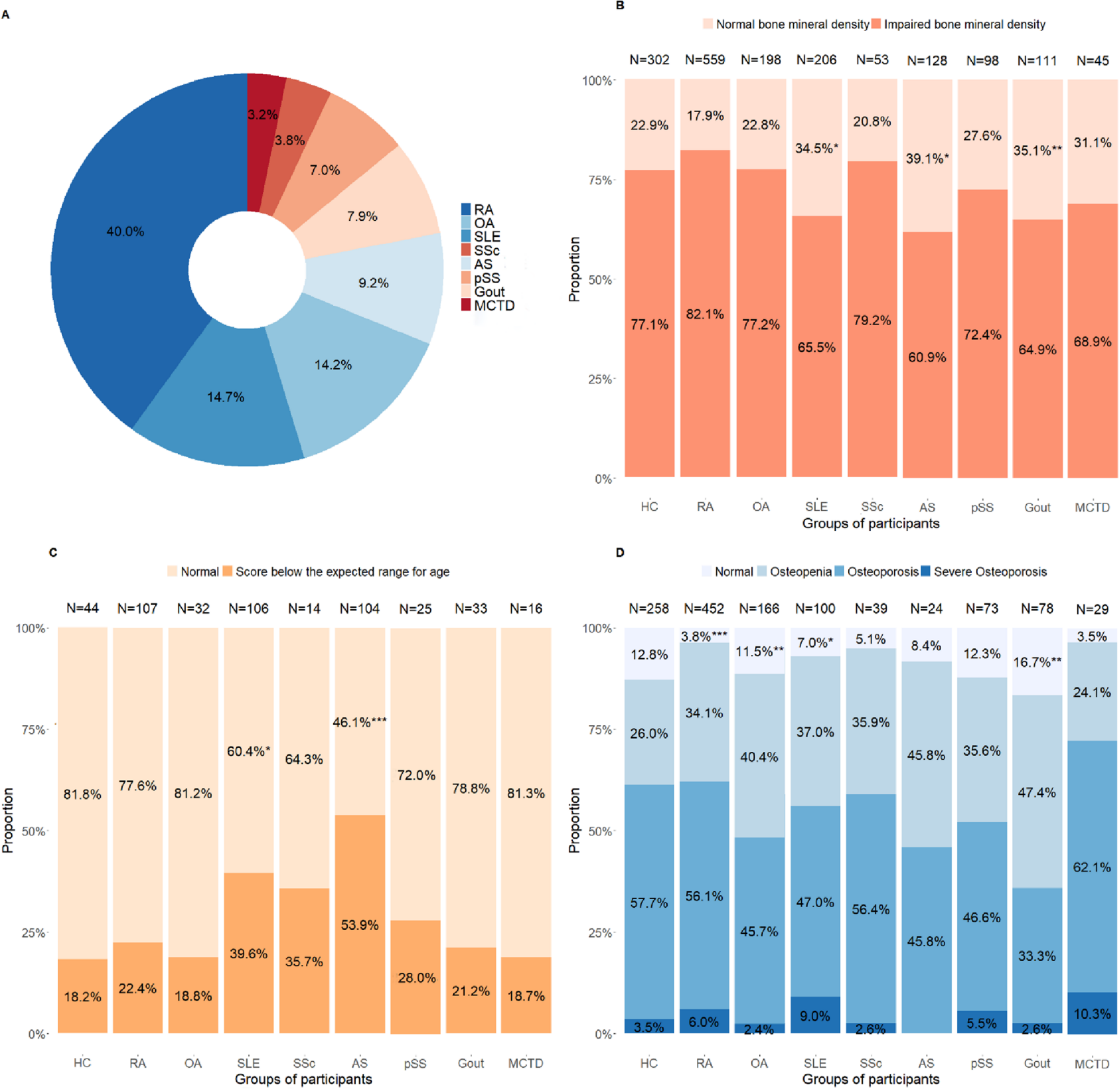
**a**: composition of rheumatic patients; **b**: comparison of impaired BMD between healthy subjects and all rheumatic patients, without age-stratification; **c**: prevalence of ‘score below the expected range for age’ in different groups; **d**: prevalence of osteopenia, osteoporosis, and severe osteoporosis in different groups. *: *P* < 0.05; **: *P* < 0.01;***: *P* < 0.001. HC: healthy controls; RA: rheumatoid arthritis; OA: osteoarthritis; SLE: systemic lupus erythematosus; SSc: systemic scleroderma; AS: ankylosing spondylitis; pSS: primary Sjogren syndrome; MCTD: mixed connective tissue disease

A total of 1398 patients and 302 healthy subjects participated in this study. RA group takes up the largest proportion of patients (40.0%), followed by SLE (14.7%) and OA (14.2%); details are shown in Fig. 1a. The basic demographic characteristics of the participants stratified by diagnosis are presented in Table 1. Age and gender compositions in some groups of patients differed from HC. The other general characteristics are shown in Table 2. Smoking and drinking are not frequent in our cohort (8.9 and 6.3%, respectively). Hypertension is the most complication (24.9%), followed by hyperuricemia (23.2%). Hypovitaminosis D is common in our cohort (68.6%).

**Table 1:** Demographic characteristics of participants

	HC	RA	***P***^***a***^	OA	***P***^***a***^	SLE	***P***^***a***^	SSc	***P***^***a***^
	***N*** = 302	***N*** = 559		***N*** = 198		***N*** = 206		***N*** = 53	
**Age, years, median [IQR]**	63.0 [53.2;72.8]	58.0 [50.0;66.0]	< 0.001	61.0 [53.0;69.8]	0.188	45.0 [35.0;54.0]	< 0.001	54.0 [45.0;59.0]	< 0.001
**Disease duration**^**b**^**, years, median [IQR]**	NA	5.6 [2.0;12.0]	NA	4.0 [1.5;10.0]	NA	3.0 [1.0;7.0]	NA	3.5 [2.0;7.0]	NA
**Age groups, years, n(%)**			< 0.001		0.008		< 0.001		0.019
< 30	19 (6.3)	10 (1.8)		2 (1.0)		37 (18.0)		2 (3.8)	
31–39	9 (3.0)	24 (4.2)		4 (2.0)		38 (18.4)		5 (9.4)	
40–49	25 (8.3)	105 (18.8)		29 (14.6)		50 (24.3)		10 (18.9)	
≥ 50	249 (82.5)	420 (75.1)		163 (82.3)		81 (39.3)		36 (67.9)	
**BMI, Kg/m**^**2**^**, mean (SD)**	22.5 (3.7)	21.9 (3.5)	0.321	24.1 (4.0)	< 0.001	22.0 (3.6)	0.085	21.8 (3.0)	0.935
**Gender, female, n(%)**	229 (75.8)	450 (80.5)	0.173	165 (83.3)	0.087	187 (90.8)	< 0.001	38 (71.7)	0.676
**Menopause status of female, n(%)**			0.01		0.277		< 0.001		0.067
Post-menopause	202 (88.2)	359 (79.8)		139 (84.2)		91 (48.7)		29 (76.3)	
Early menopause, age ≤ 45	31 (15.3)	48 (13.4)		18 (12.9)		18 (19.8)		4 (13.8)	
	**HC**	**AS**	***P***^***a***^	**pSS**	***P***^***a***^	**Gout**	***P***^***a***^	**MCTD**	***P***^***a***^
	***N*** **= 302**	***N*** **= 128**		***N*** **= 98**		***N*** **= 111**		***N*** **= 45**	
**Age, years, median [IQR]**	63.0 [53.2;72.8]	36.5 [27.0;45.2]	< 0.001	54.5 [46.0;60.0]	< 0.001	61.0 [48.0;72.0]	0.269	56.0 [46.0;62.0]	< 0.001
**Disease duration**^**b**^**, years, median [IQR]**	NA	6.5 [3.0;12.0]	NA	1.2 [0.6;2.3]	NA	6.0 [3.0;11.5]	NA	1.3 [0.5;4.5]	NA
**Age groups, years, n(%)**			< 0.001		0.003		0.006		0.003
< 30	19 (6.3)	42 (32.8)		5 (5.1)		5 (4.5)		2 (4.4%)	
31–39	9 (3.0)	30 (23.4)		10 (10.2)		7 (6.31)		2 (4.4)	
40–49	25 (8.3)	36 (28.1)		17 (17.3)		22 (19.8)		13 (28.9)	
≥ 50	249 (82.5)	20 (15.6)		66 (67.3)		77 (69.4)		28 (62.2)	
**BMI, Kg/m**^**2**^**, mean (SD)**	22.5 (3.7)	22.0 (4.2)	0.865	21.3 (2.9)	0.094	24.1 (3.3)	0.003	21.6 (3.0)	0.79
**Gender, female, n(%)**	229 (75.8)	40 (31.2)	< 0.001	91 (92.9)	0.001	18 (16.2)	< 0.001	39 (86.7)	0.197
**Menopause status of female, n(%)**			< 0.001		0.001		0.734		0.001
Post-menopause	202 (88.2)	9 (22.5)		66 (72.5)		17 (94.4)		26 (66.7)	
Early menopause, age ≤ 45	31 (15.3)	3 (33.3)		5 (7.6)		5 (29.4)		7 (26.9)	

^a^Compared with healthy controls. ^b^:correlated with age

*HC* healthy controls, *RA* rheumatoid arthritis, *OA* osteoarthritis, *SLE* systemic lupus erythematosus, *SSc* systemic scleroderma, *AS* ankylosing spondylitis, *pSS* primary Sjogren syndrome, *MCTD* mixed connective tissue disease

**Table 2:** Baseline characteristics of all patients

Characteristic	Results
**Former or current smoking, n(%)**	151 (8.9)
**Always drinking, n(%)**	107 (6.3)
**Comorbidities, n(%)**	
Diabetes Mellitus	203 (11.9)
Hypertension	424 (24.9)
Dyslipidemia	284 (16.7)
Hyperuricemia	395 (23.2)
Hypovitaminosis D	1167 (68.6)
**Complications, n(%)**	
Femoral head necrosis	28 (1.6)
Osteoporotic fracture	75 (4.4)
**Medication history, yes, n(%)**	
Former or current chronic oral Glucocorticoid therapy	713 (41.9)
NSAIDs	799 (47.0)
cDMARDs	
1 Type	261 (15.5)
2 Types	336 (20.0)
3 Types	124 (7.4)
bDMARDs	112 (6.6)
**Blood calcium level, mean (SD)**	2.6 (7.8)
**Serum phosphate level, mean (SD)**	1.3 (2.6)
**Serum creatinine level, median [IQR]**	62.0 [53.0, 75.3]
**Serum Uric acid level, n(%)**	
< 360	1068 (63.6)
360–419	249 (14.8)
420 ~ 539	243 (14.5)
≥540	118 (7.0)
**CRP, median [IQR]**	7.1 [1.5, 30.9]
**ESR, median [IQR]**	34.5 [15.0, 66.0]
**Serum 25(OH)D3 level, mean (SD)**	64.0 (26.3)
**Elevated inflammatory markers, n(%)**	
ESR	972 (63.8)
CRP	729 (47.8)

*NSAIDs* non-steroidal anti-inflammatory drugs, *cDMARDs* conventional disease-modifying anti-rheumatic drugs, *bDMARDs* biological disease-modifying anti-rheumatic drugs, *ESR* erythrocyte sedimentation rate, *CRP* c-reactive protein

### Prevalence of impaired BMD in two age-stratified population

As shown in Fig. 1b, compared with healthy subjects enrolled in our study, only patients with gout and AS are found less prevalent in impaired BMD. Both young rheumatic patients (those diagnosed with *Z*-score, 34.3% vs 18.2%, *P* = 0.045) and the elder (those diagnosed with *T*-score, 92.7% vs 87.2%, *P* = 0.017) have a statistical significance of higher prevalence of bone loss (supplement Fig. 1).

The detailed prevalence of ‘score below than expected range of age’ is shown in Fig. 1c. Patients with AS (53.9%, *P <* 0.001) and SLE (39.6%, *P* = 0.034) have a significant higher occurrence of bone loss, compared with HC (18.2%).

Prevalence of varying degrees of bone loss among men aged ≥50 and postmenopausal women is shown in Fig. 1d. It was obviously higher in patients with RA (*P for trend* < 0.001), OA (*P for trend =* 0.02) and SLE (*P for trend* = 0.011), but lower in gout (*P for trend =* 0.001) compared with healthy peers.

### The odds ratio for bone loss in rheumatic patients

**Fig. 2:**
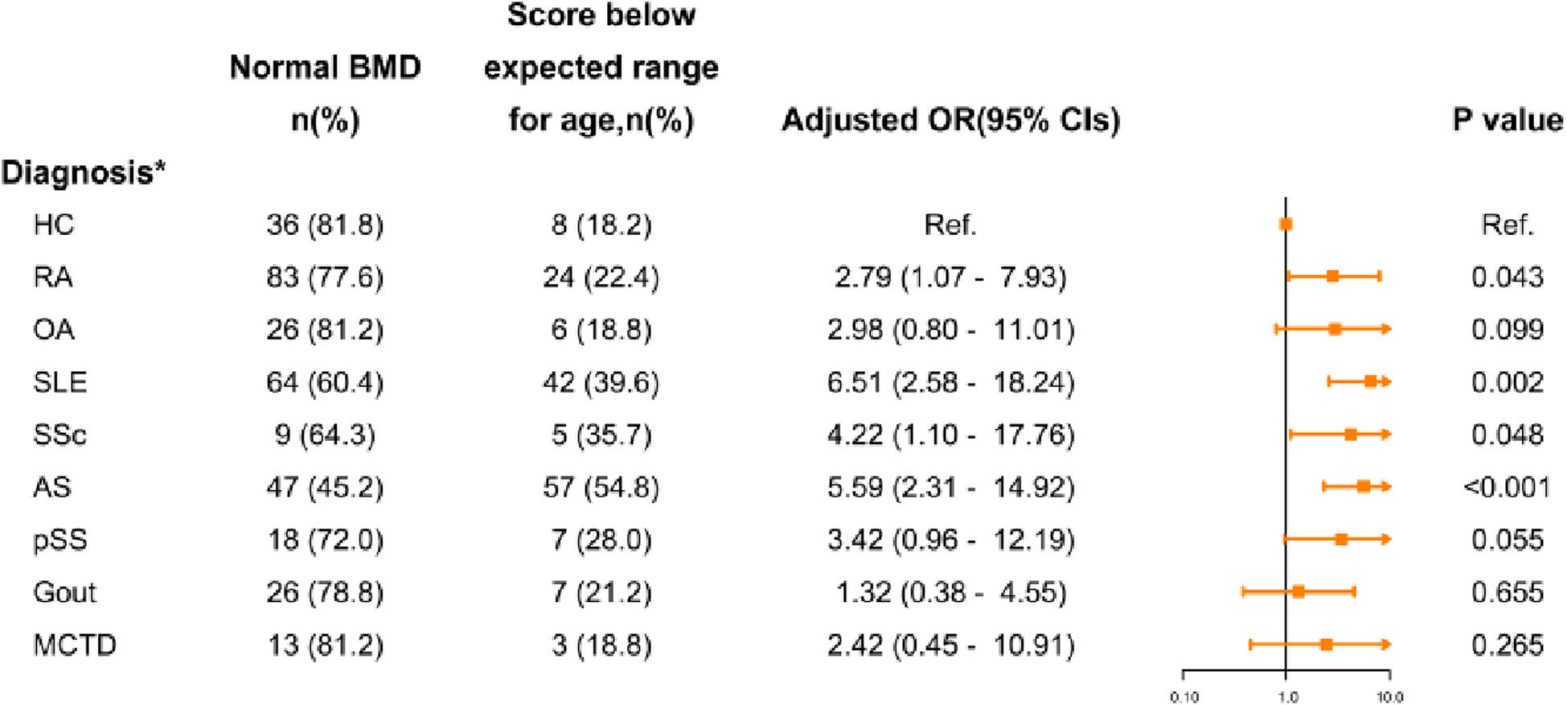
Odds ratio of ‘Score below expected range for age’ in rheumatic patients scored by *Z*-score. *: age-, gender-, BMI- and GC therapy- adjusted. *HC*: healthy controls; *RA*: rheumatoid arthritis; *OA*: osteoarthritis;* SLE*: systemic lupus erythematosus;* SSc*: systemic scleroderma; *AS*: ankylosing spondylitis; *pSS*: primary Sjogren syndrome; *MCTD*: mixed connective tissue disease

**Fig. 3:**
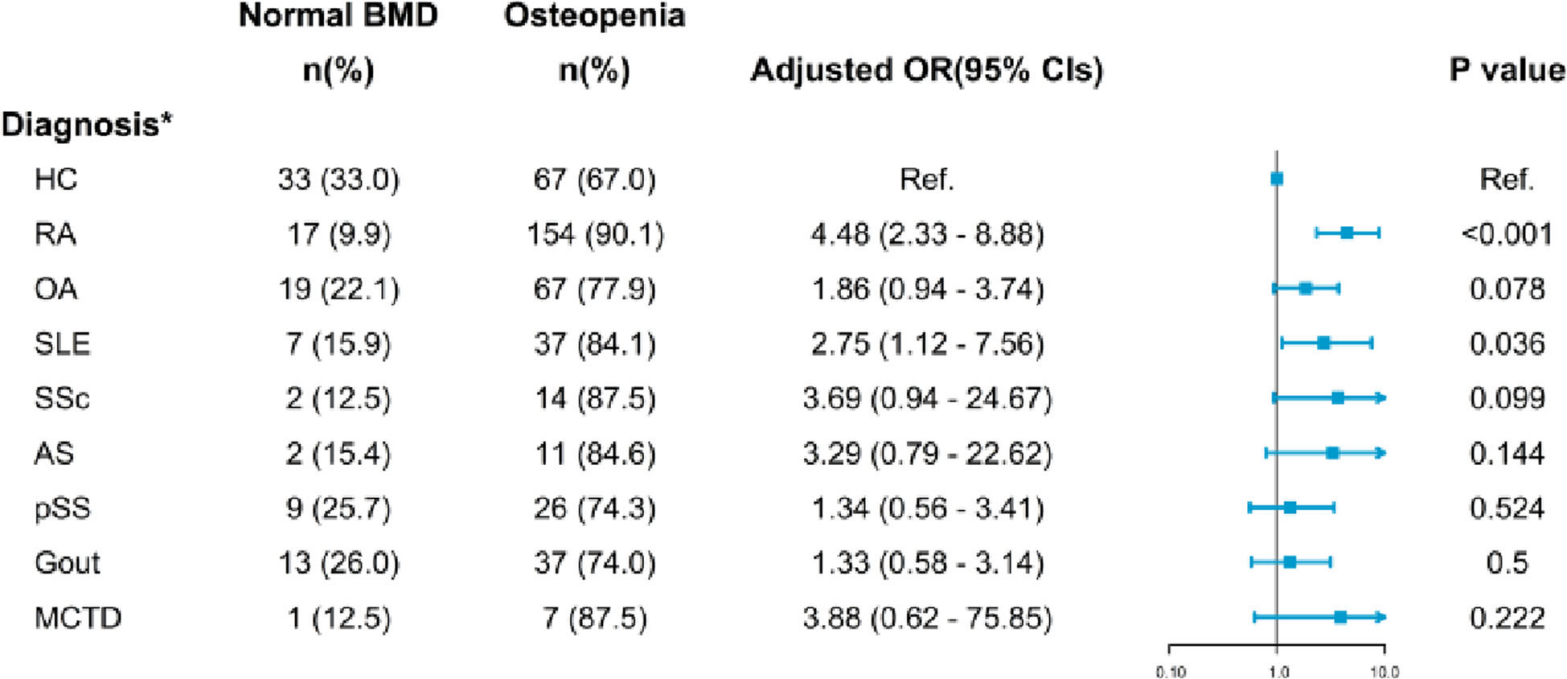
Odds ratio ofosteopenia in rheumatic patients scored by *T*-score. *: age-, gender-, BMI- and GC therapy- adjusted.* HC*: healthy controls; *RA*: rheumatoid arthritis; *OA*: osteoarthritis; *SLE*: systemic lupus erythematosus;* SSc*: systemic scleroderma; *AS*: ankylosing spondylitis; _pSS_: primary Sjogren syndrome; *MCTD*: mixed connective tissue disease

**Fig. 4:**
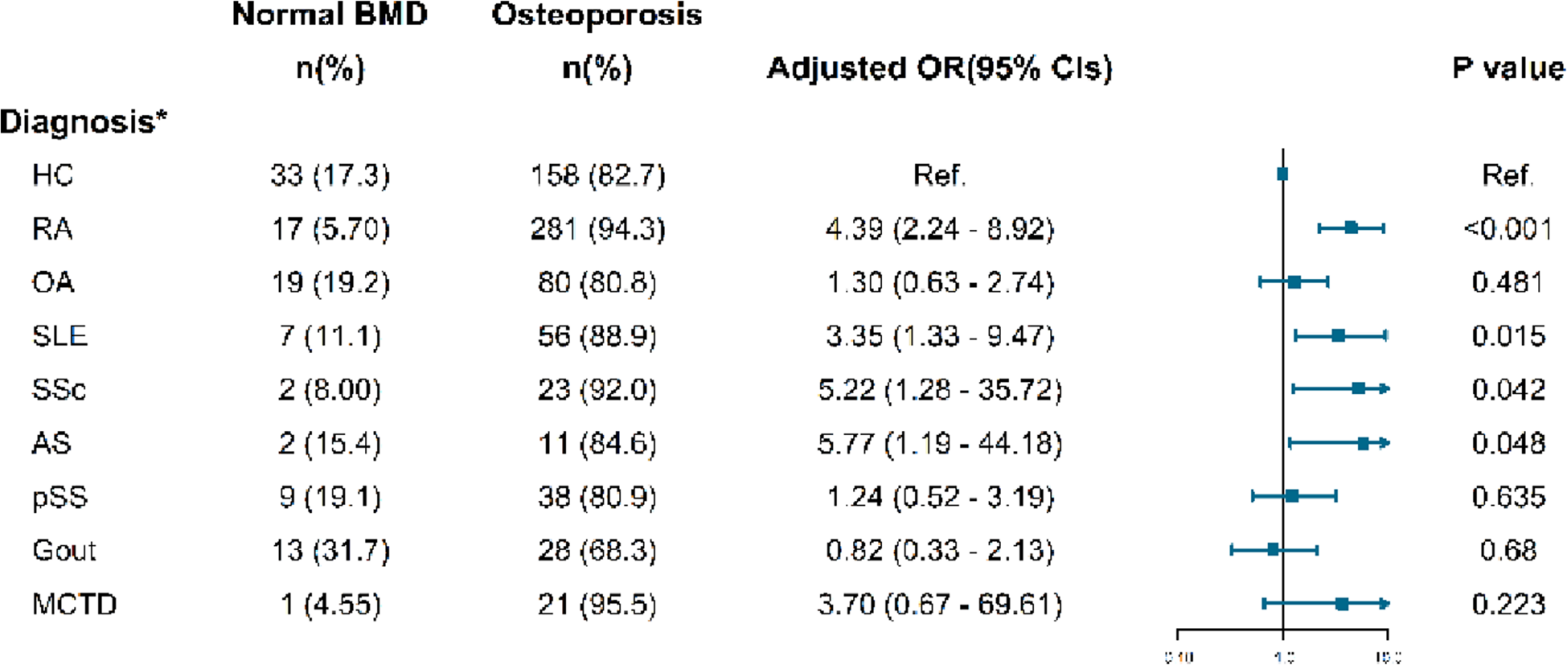
Odds ratio of osteoporosis in rheumatic patients scored by *T*-score. *: age-, gender-, BMI- and GC therapy- adjusted. *HC*: healthy controls; *RA*: rheumatoid arthritis; *OA*: osteoarthritis; *SLE*: systemic lupus erythematosus; *SSc*: systemic scleroderma; *AS*: ankylosing spondylitis; *pSS*: primary Sjogren syndrome; *MCTD*: mixed connective tissue disease

Figures 2, 3 and 4 shows the relationships among the impaired BMD and variables in rheumatic patients compared with the healthy group, using an age-, gender-, BMI- and GC therapy- adjusted logistic regression model. Results showed young patients with SLE gained the highest risk, reached about 6.5-fold, and followed by AS (5.6-fold). In patients classified by *T*-score, namely men aged 50 or over and postmenopausal women, RA (4.5-fold) and SLE (2.8-fold) patients have both greater risk of osteopenia and osteoporosis. Patients with AS and SSc obtained the highest risk of osteoporosis, similarly 5 times higher. Notwithstanding, in patients with OA, pSS, gout, and MCTD, no significant risk of any sort of bone loss was found.

We next aimed to explore risk factors that account for bone loss among rheumatic patients, stratified by age groups (i.e. scoring methods) and GC usage. 207 of young patients (a total of 423 young patients) were reported with GC treatment. The prevalence of ‘score below the expected range for age’ in GC and non-GC group is 37.5% vs. 30.9% (*P* = 0.186). Details are shown in Table 3. In those without using GC, hypovitaminosis D is a risk factor for BMD; moreover, increased age, female and BMI are both relative to bone loss in two subgroups.

**Table 3:** Odd ratios of variables in rheumatic patients with ‘score below the expected range for age’

Variables	‘Score below the expected range for age’	
	OR (95% CIs)	***P***
**Former or current chronic GC usage**	*N* = 64	
**Age**^**a**^	0.96 [0.93;0.99]	0.014
**Disease duration**^**b**^	1.07 [1.01;1.13]	0.025
**BMI, Kg/m**^**2**^		
< 18.5 (underweight)	3.57 [1.55;8.43]	0.003
18.5–23.9 (normal)	Ref.	Ref.
24–27.9 (overweight)	0.83 [0.34;1.89]	0.674
≥ 28(obese)	4.99 [1.38;20.9]	0.014
**Gender, compared with male**		
Female	0.38 [0.17;0.83]	0.015
**Medical history**		
Diabetes Mellitus	1.38 [0.26;6.05]	0.68
Hypertension	2.33 [0.61;8.96]	0.211
Dyslipidemia	0.86 [0.26;2.44]	0.79
Hyperuricemia	1.36 [0.58;3.03]	0.466
**Former or current smokers**	0.86 [0.17;3.15]	0.825
**Regular drinking**	0.49 [0.02;3.26]	0.503
**Hypovitaminosis D**	1.23 [0.65;2.37]	0.525
**CRP elevated**	1.76 [0.97;3.23]	0.063
**ESR elevated**	1.57 [0.83;3.08]	0.17
**Non-GC usage**	*N* = 81	
**Age**^**a**^	0.94 [0.92;0.97]	< 0.001
**Disease duration**^**b**^	1.00 [0.95;1.04]	0.866
**BMI, Kg/m**^**2**^		
< 18.5 (underweight)	3.53 [1.61;8.07]	0.001
18.5–23.9 (normal)	Ref.	Ref.
24–27.9 (overweight)	0.81 [0.39;1.65]	0.568
≥ 28(obese)	0.09 [0.00;0.45]	0.001
**Gender, compared with male**		
Female	0.39 [0.22;0.68]	0.001
**Medical history**		
Diabetes Mellitus	0.22 [0.01;1.28]	0.102
Hypertension	0.65 [0.22;1.69]	0.389
Dyslipidemia	0.94 [0.37;2.21]	0.883
Hyperuricemia	1.80 [0.96;3.38]	0.067
**Former or current smokers**	1.50 [0.64;3.46]	0.342
**Regular drinking**	0.58 [0.18;1.60]	0.306
**Hypovitaminosis D**	1.91 [1.03;3.62]	0.039
**CRP elevated**	1.13 [0.65;1.97]	0.676
**ESR elevated**	0.86 [0.49;1.51]	0.591

^a^continuous variable; ^b^continuous variable and correlated with age

Four hundred sixty-six of 922 old patients were reported positive GC using history. However, both GC (94.2%) and non-GC group (91.0%, *P* = 0.083) have high prevalence of impaired BMD in older rheumatic patients. In these patients who scored with *T*-score and using GC, age and longer disease duration, female, overweight and obesity, hypovitaminosis D are both associated with osteopenia and osteoporosis (shown in Table 4). Dyslipidemia and hyperuricemia were found a protective factor for BMD. Compare with those with GC therapy, patients in non-GC group did not find disease duration and dyslipidemia have a significant influence on BMD, but regular cigarette or alcohol intake were found a protective factor in these patients.

**Table 4:** Odd ratios of variables in rheumatic patients with osteopenia and osteoporosis

Variables	Osteopenia		Osteoporosis	
	OR (95% CIs)	***P***	OR (95% CIs)	***P***
**Former or current chronic GC therapy**	*N* = 163		*N* = 276	
**Age**^**a**^	1.07 [1.03;1.11]	0.001	1.13 [1.08;1.18]	< 0.001
**Disease duration**^**b**^	1.06 [1.00;1.12]	0.033	1.05 [1.00;1.11]	0.052
**BMI, Kg/m**^**2**^				
< 18.5 (underweight)	1.74 [0.44;12.6]	0.469	2.98 [0.83;20.7]	0.102
18.5–23.9 (normal)	Ref.	Ref.	Ref.	Ref.
24–27.9 (overweight)	0.54 [0.26;1.11]	0.092	0.30 [0.15;0.61]	0.001
≥ 28(obese)	0.55 [0.18;1.93]	0.329	0.17 [0.05;0.65]	0.011
**Gender, compared with male**				
Female	3.08 [1.55;6.15]	0.001	3.52 [1.83;6.74]	< 0.001
**Menopause status**				
Post-menopause	Ref.	Ref.	Ref.	Ref.
Early menopause, age ≤ 45	0.96 [0.32;3.65]	0.943	1.09 [0.39;4.02]	0.877
**Medical history**				
Diabetes Mellitus	0.84 [0.34;2.28]	0.709	0.71 [0.31;1.88]	0.471
Hypertension	1.31 [0.62;3.01]	0.492	1.17 [0.57;2.63]	0.675
Dyslipidemia	0.29 [0.13;0.66]	0.003	0.45 [0.22;0.93]	0.031
Hyperuricemia	0.58 [0.27;1.30]	0.178	0.42 [0.20;0.92]	0.032
**Former or current smokers**	0.42 [0.16;1.15]	0.089	0.44 [0.19;1.12]	0.083
**Regular drinking**	0.48 [0.13;1.97]	0.284	0.65 [0.22;2.40]	0.477
**Hypovitaminosis D**	2.49 [1.29;4.90]	0.007	3.95 [2.09;7.58]	< 0.001
**CRP elevation**	1.15 [0.60;2.22]	0.673	1.60 [0.85;2.99]	0.143
**ESR elevation**	1.36 [0.68;2.67]	0.374	2.49 [1.27;4.80]	0.009
**Non-GC therapy**	*N* = 176		*N* = 239	
**Age**^**a**^	1.12 [1.09;1.16]	< 0.001	1.16 [1.12;1.20]	< 0.001
**Disease duration**^**b**^	1.02 [0.98;1.06]	0.311	1.02 [0.99;1.06]	0.128
**BMI, Kg/m**^**2**^				
< 18.5 (underweight)	0.86 [0.25;3.54]	0.826	2.04 [0.74;7.35]	0.178
18.5–23.9 (normal)	Ref.	Ref.	Ref.	Ref.
24–27.9 (overweight)	0.70 [0.40;1.23]	0.217	0.29 [0.16;0.51]	< 0.001
≥ 28(obese)	0.54 [0.26;1.14]	0.104	0.29 [0.14;0.60]	0.001
**Gender, compared with male**				
Female	1.02 [0.48;2.09]	0.949	9.34 [5.51;16.2]	< 0.001
**Menopause status**				
Post-menopause	Ref.	Ref.	Ref.	Ref.
Early menopause, age ≤ 45	1.70 [0.43;12.3]	0.485	1.09 [0.39;4.02]	0.877
**Medical history**				
Diabetes Mellitus	0.97 [0.50;1.93]	0.921	1.02 [0.55;1.99]	0.939
Hypertension	1.58 [0.92;2.75]	0.098	1.17 [0.69;2.00]	0.566
Dyslipidemia	0.87 [0.47;1.64]	0.651	0.60 [0.33;1.13]	0.114
Hyperuricemia	0.66 [0.37;1.16]	0.146	0.54 [0.31;0.93]	0.027
**Former or current smokers**	0.33 [0.16;0.66]	0.002	0.35 [0.18;0.66]	0.001
**Regular drinking**	0.29 [0.13;0.61]	0.001	0.24 [0.12;0.51]	< 0.001
**Hypovitaminosis D**	0.88 [0.51;1.50]	0.639	1.02 [0.60;1.69]	0.95
**CRP elevation**	0.77 [0.46;1.26]	0.292	0.78 [0.48;1.25]	0.294
**ESR elevation**	0.83 [0.49;1.40]	0.492	1.48 [0.87;2.48]	0.146

^a^continuous variable; ^b^continuous variable and correlative with age

## Discussion

### Prevalence of bone loss and odd ratios

In this multi-central, cross-sectional study with age-stratification, we compared the frequency and odd ratios of reduced BMD in all rheumatic patients with healthy counterparts, and examined risk factors for bone loss in patients, aimed to help prevent and efficiently treat less-heeded bone loss. No contradiction was found in our study on the prevalence of bone loss with previous literature for some rheumatic diseases: for RA patients, the prevalence of ‘score below the expected range for age’ was reported 7.8% ~ 18% [5, 32], and osteopenia and osteoporosis was 46.8% ~ 55.7% [25, 32]; a retrospective study in Spain in 2010 showed a high prevalence of osteopenia (average 36.9%) among 105 female patients with SLE [33]. Bone loss was found in 5% ~ 44% patients with AS [11]. The risk of OP in SSc was reported closely analogous to RA [24], and the occurrence of OP was 51.1% [34].

For increasing the comparability, multivariate logistic regression analyses found the adjusted odds ratios of ‘score below the expected range for age’ in patients with SLE and AS gain the highest, 6.5 times and 5.6 times higher risk, respectively. Patients with RA and SLE achieved a higher risk of osteopenia, achieving 4.5-fold and 2.8-fold respectively. Moreover, the strongest association with osteoporosis was found in AS, reaching 5.8 times higher.

OA, pSS, gout, and MCTD were not discovered related to higher risk of bone loss in our study. Except for the small sample size, a plausible scenario could be in the follows. OA is an age strongly-related degenerative disease, and BMI has opposing effect on OA and OP; also, local inflammation caused by mechanical injury, rather than systemic one caused by autoimmunology, is its salient feature [35]. Whether pSS would gain higher prevalence of OP or osteopenia is still uncertain [12], and in present study were mostly in early-onset and untreated. That might be another reason why risk of bone loss in these patients did not increase. A protective effect of uric acid (UA) on lumbar spine BMD has been reported in male patients [36] and hypothesized its potent antioxidant effect or via its interaction with the vitamin D/parathyroid hormone pathway [37], but high levels of serum UA (sUA) could cause oxidative stress and microinflammation as a pro-oxidant [38]; the role that high sUA/gout plays in OPF is also paradoxical [39–41]. In our study, likewise, hyperuricemia showed a positive effect on OP.

### Risk factors for bone loss in different age groups of rheumatic patients

The well-known association [1–3] of elder age, female and underweight (BMI < 18.5 Kg/m^2^) were also found associated with OP in our study, similar to the reported [19, 25, 42]. But the contrast was found in ‘score below the expected range for age’. It might be attributed to more than half of AS patients were young male (68.8%) in our study, who were strongly related to impaired BMD, and in female patients, estrogen has direct effects on osteocytes, osteoclasts, and osteoblasts, leading to inhibition of bone resorption and maintenance of bone formation [3, 43]. Obesity was found even as a protective factor for BMD, as reported befor e[44]. Dyslipidemia was found a protective factor for osteopenia and osteoporosis, probably it is one of the results of obesity; the association between lipid profiles and osteoporosis is still uncertain [45]. Post-menopause is well-documented risk factor for OP, owing to low level of estrogen, and 3.5 times (in those with GC) and 9.3 times (in those without GC) higher risk than male peers were found in our study.

Disease duration is correlative with age and partially reflecting the therapeutic period of GC. Long-term GC therapy and high cumulative dose have been proved to be strongly related to OP and fragile fracture [16, 46]. In a previous South Korean study [5] showed that evaluated cumulative GC dose did not correlate with reduced BMD in different detective sites but those who had a history of taking GCs. Likewise, our results showed a higher risk of bone loss upon chronic GC therapeutic history.

In addition, we found regular alcohol and cigarette intake had a protective effect upon osteopenia and osteoporosis in older patients without GC therapy, but there were still insufficient samples and undetailed daily and period of consumption in our study. A British study [47] on 651 young males showed that moderate alcohol intake perhaps benefited to BMD, but smoking was detrimental, even short duration of smoking.

This study has limitations. First, we could not exclude the possibility of patient selection bias, because the 4 centers participating in this study were tertiary referral centers in Southern China. Second, in-patients with higher disease activity and longer disease duration, and healthy subjects with higher traditional risks are more willing to receive BMD examinations because hospitalized patients can reimburse the fee BMD test cost. Therefore, our study revealed a higher prevalence of OP than the previous reported. Third, cross-sectional studies could not control baseline as balanced as prospective study; it could not reveal dynamic changes with time, neither. We are looking forward to a long-term follow-up study on the BMD change, and to demonstrate potential risk factors of bone loss in rheumatic patients.

## Conclusions

Young patients with AS and SLE have a significant higher occurrence of bone loss, and older patients with RA, OA and SLE had higher prevalence than healthy counterparts. SLE, RA, SSc and AS were founded significant higher risks to develop into bone loss after adjustment. Age, BMI, and gender were commonly-associated with bone loss in all age-stratified rheumatic patients. These findings were not markedly different from those of previous studies.

## Supplementary information

**Additional file 1.**

**Additional file 2: Figure S1.** Prevalence of impaired BMD in two age groups.

## Data Availability

The data that support the findings of this study are available from hospital informational system of 1) Third Affiliated Hospital of Sun Yat-sen University, 2) Ganzhou Municipal Hospital, Ganzhou, Jiangxi Province, China, 3) Fujian Provincial Hospital, Fuzhou, Fujian Province, China, 4) Second Affiliated Hospital of Shantou University Medical College. Data are presented within additional supporting files, but identifying patient data are not shared.

## References

[1] Lane NE (2006). Epidemiology, etiology, and diagnosis of osteoporosis. Am J Obstet Gynecol.

[2] Hossein-Nezhad A, Holick MF (2013). Vitamin D for health: a global perspective. Mayo Clin Proc.

[3] Black DM, Rosen CJ (2016). Postmenopausal osteoporosis. N Engl J Med.

[4] Sapir-koren R, Livshits G (2017). Postmenopausal osteoporosis in rheumatoid arthritis : the estrogen de fi ciency-immune mechanisms link. Bone..

[5] Lee SG, Park YE, Park SH, Kim TK, Choi HJ, Lee SJ (2012). Increased frequency of osteoporosis and BMD below the expected range for age among south Korean women with rheumatoid arthritis. Int J Rheum Dis.

[6] Avouac J, Koumakis E, Toth E, Meunier M, Maury E, Kahan A (2012). Increased risk of osteoporosis and fracture in women with systemic sclerosis: a comparative study with rheumatoid arthritis. Arthritis Care Res.

[7] Bultink IEM, Lems WF (2016). Lupus and fractures. Curr Opin Rheumatol.

[8] Carli L, Tani C, Spera V, Vagelli R, Vagnani S, Mazzantini M (2016). Risk factors for osteoporosis and fragility fractures in patients with systemic lupus erythematosus. Lupus Sci Med.

[9] Chen ZY, Kok VC, Hung GD, Horng JT, Kuo JT, Wang MN (2018). Gout as a risk factor for osteoporosis: epidemiologic evidence from a population-based longitudinal study involving 108,060 individuals. Osteoporos Int.

[10] Kim YH, Lee JS, Park JH (2018). Association between bone mineral density and knee osteoarthritis in Koreans: the fourth and fifth Korea National Health and nutrition examination surveys. Osteoarthr Cartil.

[11] Klingberg E, Lorentzon M, Mellström D, Geijer M, Göthlin J, Hilme E (2012). Osteoporosis in ankylosing spondylitis - prevalence, risk factors and methods of assessment. Arthritis Res Ther.

[12] Gravani F, Papadaki I, Antypa E, Nezos A, Masselou K, Ioakeimidis D (2015). Subclinical atherosclerosis and impaired bone health in patients with primary Sjogren's syndrome: prevalence, clinical and laboratory associations. Arthritis Res Ther.

[13] Bodolay E, Bettembuk P, Balogh Á, Szekanecz Z (2003). Osteoporosis in mixed connective tissue disease. Clin Rheumatol.

[14] Gough AK, Lilley J, Eyre S, Holder RL, Emery P (1994). Generalised bone loss in patients with early rheumatoid arthritis. Lancet..

[15] Gough A, Sambrook P, Devlin J, Huissoon A, Njeh C, Robbins S (1998). Osteoclastic activation is the principal mechanism leading to secondary osteoporosis in rheumatoid arthritis. J Rheumatol.

[16] Buckley L, Guyatt G, Fink HA, Cannon M, Grossman J, Hansen KE (2017). American College of Rheumatology Guideline for the prevention and treatment of glucocorticoid-induced osteoporosis. Arthritis Rheumatol (Hoboken, NJ).

[17] Axelsson KF, Nilsson AG, Lorentzon M (2017). Alendronate and Hip Fracture in Patients Using Glucocorticoids—Reply. JAMA.

[18] Rentero ML, Amigo E, Chozas N, Fernández Prada M, Silva-Fernández L, Abad Hernandez MA (2015). Prevalence of fractures in women with rheumatoid arthritis and/or systemic lupus erythematosus on chronic glucocorticoid therapy epidemiology of musculoskeletal disorders. BMC Musculoskelet Disord.

[19] Wang DM, Zeng QY, Chen SB, Gong Y, Hou ZD, Xiao ZY (2015). Prevalence and risk factors of osteoporosis in patients with ankylosing spondylitis: a 5-year follow-up study of 504 cases. Clin Exp Rheumatol.

[20] Papageorgiou M, Dolan E, Elliott-Sale KJ, Sale C (2018). Reduced energy availability: implications for bone health in physically active populations. Eur J Nutr.

[21] Kerr C, Bottomley C, Shingler S, Giangregorio L, de Freitas HM, Patel C (2017). The importance of physical function to people with osteoporosis. Osteoporos Int.

[22] Compston J (2018). Glucocorticoid-induced osteoporosis: an update. Endocrine..

[23] Cramarossa G, Urowitz MB, Su J, Gladman D, Touma Z (2017). Prevalence and associated factors of low bone mass in adults with systemic lupus erythematosus. Lupus..

[24] Kilic G, Kilic E, Akgul O, Ozgocmen S (2016). Increased risk for bone loss in women with systemic sclerosis: a comparative study with rheumatoid arthritis. Int J Rheum Dis.

[25] Lee JH, Sung YK, Choi CB, Cho SK, Bang SY, Choe JY (2016). The frequency of and risk factors for osteoporosis in Korean patients with rheumatoid arthritis. BMC Musculoskeletal Disord..

[26] Chen P, Li Z, Hu Y (2016). Prevalence of osteoporosis in China: a meta-analysis and systematic review. BMC Public Health.

[27] Appropriate body-mass index for Asian populations and its implications for policy and intervention strategies. Lancet. 2004;363(9403):157–63. 10.1016/S0140-6736(03)15268-3 14726171

[28] Wildman RP, Gu D, Reynolds K, Duan X, He J (2004). Appropriate body mass index and waist circumference cutoffs for categorization of overweight and central adiposity among Chinese adults. Am J Clin Nutr.

[29] World Health Organization. Prevention and management of osteoporosis. World Health Organization technical report series. 2003(921):1–164. 15293701

[30] Hans D, Downs RW, Duboeuf F, Greenspan S, Jankowski LG, Kiebzak GM (2006). Skeletal sites for osteoporosis diagnosis: the 2005 ISCD official positions. J Clin Densitometry.

[31] Subirana I, Sanz H, Vila J (2014). Building Bivariate Tables: The compareGroups Package for R. J Stat Software.

[32] Oelzner P, Schwabe A, Lehmann G, Eidner T, Franke S, Wolf G (2008). Significance of risk factors for osteoporosis is dependent on gender and menopause in rheumatoid arthritis. Rheumatol Int.

[33] Monte TS, Ruiz JP, Torrente-Segarra V, Mojal S, Padró I, Carbonell J (2013). Prevalence of osteopenia and osteoporosis in women with systemic lupus erythematosus. Ann Rheum Dis.

[34] Souza RB, Borges CT, Takayama L, Aldrighi JM, Pereira RM (2006). Systemic sclerosis and bone loss: the role of the disease and body composition. Scand J Rheumatol.

[35] Geusens PP, van den Bergh JP (2016). Osteoporosis and osteoarthritis: shared mechanisms and epidemiology. Curr Opin Rheumatol.

[36] Xiao J, Chen W, Feng X, Liu W, Zhang Z, He L (2017). Serum uric acid is associated with lumbar spine bone mineral density in healthy Chinese males older than 50 years. Clin Interv Aging.

[37] Chen W, Roncal-Jimenez C, Lanaspa M, Gerard S, Chonchol M, Johnson RJ (2014). Uric acid suppresses 1 alpha hydroxylase in vitro and in vivo. Metab Clin Exp.

[38] Kang DH, Ha SK (2014). Uric acid puzzle: dual role as anti-oxidantand pro-oxidant. Electrolyte Blood Pressure.

[39] Wang Y, Zhou R, Zhong W, Hu C, Lu S, Chai Y (2018). Association of gout with osteoporotic fractures. Int Orthop.

[40] Kim SC, Paik JM, Liu J, Curhan GC, Solomon DH (2017). Gout and the risk of non-vertebral fracture. J Bone Miner Res.

[41] Kim BJ, Baek S, Ahn SH, Kim SH, Jo MW, Bae SJ (2014). Higher serum uric acid as a protective factor against incident osteoporotic fractures in Korean men: a longitudinal study using the National Claim Registry. Osteoporos Int.

[42] Omair MA, Pagnoux C, McDonald-Blumer H, Johnson SR (2013). Low bone density in systemic sclerosis. A systematic review. J Rheumatol.

[43] Henes M, Froeschlin J, Taran FA, Brucker S, Rall KK, Xenitidis T (2015). Ovarian reserve alterations in premenopausal women with chronic inflammatory rheumatic diseases: impact of rheumatoid arthritis, Behcet's disease and spondyloarthritis on anti-Mullerian hormone levels. Rheumatology (Oxford).

[44] Alay I, Kaya C, Cengiz H, Yildiz S, Ekin M, Yasar L (2020). The relation of body mass index, menopausal symptoms, and lipid profile with bone mineral density in postmenopausal women. Taiwan J Obstet Gynecol.

[45] Chen YY, Wang WW, Yang L, Chen WW, Zhang HX (2018). Association between lipid profiles and osteoporosis in postmenopausal women: a meta-analysis. Eur Rev Med Pharmacol Sci.

[46] Hartmann K, Koenen M, Schauer S, Wittig-Blaich S, Ahmad M, Baschant U (2016). Molecular actions of glucocorticoids in cartilage and bone during health, disease, and steroid therapy. Physiol Rev.

[47] Eleftheriou KI, Rawal JS, James LE, Payne JR, Loosemore M, Pennell DJ (2013). Bone structure and geometry in young men: the influence of smoking, alcohol intake and physical activity. Bone..

