# Supplementary figures and images for: Prevalence and risk factors for bone loss in Southern Chinese with rheumatic diseases
Source: BMC Musculoskelet Disord. 2020 Jun 30;21:416. doi: 10.1186/s12891-020-03403-1 (PMC7329556; doi:10.1186/s12891-020-03403-1)

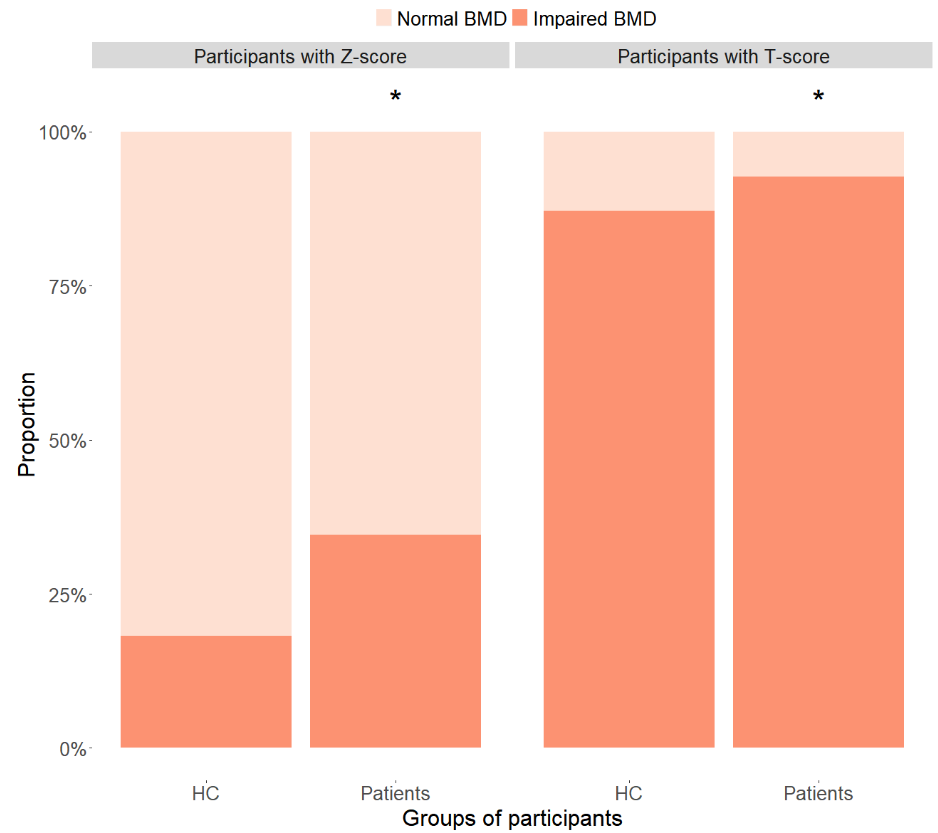


92.7%

87.2%

18.2%

34.3%

Supplementary Fig 1 Prevalence of impaired BMD in two age groups

Supplement: Supplementary file 2 — Additional file 2: Figure S1. Prevalence of impaired BMD in two age groups. [file 12891_2020_3403_MOESM2_ESM.docx]
